# Development of a Digital Support Application With Evidence-Based Content for Sustainable Return to Work for Persons With Chronic Pain and Their Employers: User-Centered Agile Design Approach

**DOI:** 10.2196/33571

**Published:** 2022-03-14

**Authors:** Christina Turesson, Gunilla Liedberg, Mathilda Björk

**Affiliations:** 1 Division of Prevention, Rehabilitation and Community Medicine Department of Health, Medicine and Caring Sciences Linköping University Norrköping Sweden; 2 Pain and Rehabilitation Centre Department of Health, Medicine and Caring Sciences Linköping University Linköping Sweden

**Keywords:** agile design process, chronic pain, digital support, eHealth, return to work, self-management, smartphone apps, user-centered design, mobile phone

## Abstract

**Background:**

Persons with chronic pain experience a lack of support after completing rehabilitation and the responsibility for the return-to-work (RTW) process is taken over by the employer. In addition, employers describe not knowing how to support their employees. Smartphone apps have been increasingly used for self-management, but there is a lack of available eHealth apps with evidence-based content providing digital support for persons with chronic pain and their employers when they return to work.

**Objective:**

This study aims to describe the development of a digital support application with evidence-based content that includes a biopsychosocial perspective on chronic pain for sustainable RTW for persons with chronic pain and their employers (SWEPPE [Sustainable Worker Digital Support for Persons With Chronic Pain and Their Employers]).

**Methods:**

A user-centered agile design approach was applied. The multidisciplinary project team consisted of health care researchers, a user representative, and a software team. A total of 2 reference groups of 7 persons with chronic pain and 4 employers participated in the development process and usability testing. Mixed methods were used for data collection. The design was revised using feedback from the reference groups. The content of SWEPPE was developed based on existing evidence and input from the reference groups.

**Results:**

The reference groups identified the following as important characteristics to include in SWEPPE: keeping users motivated, tracking health status and work situation, and following progress. SWEPPE was developed as a smartphone app for the persons with chronic pain and as a web application for their employers. SWEPPE consists of six modules: the action plan, daily self-rating, self-monitoring graphs, the coach, the library, and shared information with the employer. The employers found the following functions in SWEPPE to be the most useful: employees’ goals related to RTW, barriers to RTW, support wanted from the employer, and the ability to follow employees’ progress. The persons with chronic pain found the following functions in SWEPPE to be the most useful: setting a goal related to RTW, identifying barriers and strategies, and self-monitoring. Usability testing revealed that SWEPPE was safe, useful (ie, provided relevant information), logical, and easy to use with an appealing interface.

**Conclusions:**

This study reports the development of a digital support application for persons with chronic pain and their employers. SWEPPE fulfilled the need of support after an interdisciplinary pain rehabilitation program with useful functions such as setting a goal related to RTW, identification of barriers and strategies for RTW, self-monitoring, and sharing information between the employee and the employer. The user-centered agile design approach contributed to creating SWEPPE as a relevant and easy-to-use eHealth intervention. Further studies are needed to examine the effectiveness of SWEPPE in a clinical setting.

## Introduction

### Background

Chronic musculoskeletal pain, which affects 10% to 20% of the European population and negatively impacts functioning, quality of life, and the ability to work, comes with significant individual and societal expenses, including costs associated with sick leave and loss of productivity [1-6]. A recently published interview study [7] showed that persons with chronic pain experienced a lack of support after completing a rehabilitation program when responsibility for the return-to-work (RTW) process was taken over by the employer. In addition, the employers reported lacking knowledge on how to support their employees’ RTW and requested more knowledge about how chronic pain might affect work status and the needs and challenges their employees with chronic pain might experience [7]. For a successful RTW, employers need to effectively collaborate, communicate, and negotiate with their employees, all interactions that require good listening skills [8,9]. In general, barriers to RTW for persons with chronic pain include lack of workplace support, lack of relationships with supervisors and coworkers, and inability to find the right fit between a person’s physical abilities and job tasks [10,11]. A smartphone app could be used to deal with the above challenges, improve the rehabilitation process, and counteract passivity by increasing interaction between the employer and the employee [12], leading to a shared decision-making model [13] used in work rehabilitation to increase a successful outcome in the RTW process.

Digital support (web-based applications or smartphone apps) is a growing intervention for persons with chronic pain and a useful tool for quality of learning [14-16]. The strengths of digital interventions include evidence-based content; possibility for daily registrations of health aspects; simple design with short, easily readable texts [17]; and reading about other people’s experiences [18]. Typically, self-management includes providing knowledge and education about the condition (including its consequences) and self-assessment of health [17,19,20]. This can contribute to the individuals’ learning about their own capacity [21,22], which can lead to an increased sense of control and motivation for continued self-management [23].

Digital applications can be valuable tools for persons with chronic pain, especially when used in an outclinic setting [24], and can reduce pain and disability [25,26]. Despite these positive effects, research has reported limitations related to the low overall quality of smartphone apps for chronic pain and the lack of rigorous assessment of their effectiveness [27,28]. Therefore, combining evidence-based concepts with stakeholder involvement in the development of eHealth interventions is highly important [15,27]. The key elements of user-centered design (UCD) approaches include stakeholder involvement, iterative design and development, user stories, user personas, interviews, prototyping, and usability testing to identify and fulfill the users’ needs and requirements [29-33]. To manage challenges such as incorrect clinical or user context or flaws in evaluation [34], researchers need to use a multidisciplinary development approach, continuous and systematic evaluation, and robust evaluation methods [35].

### Objectives

Web-based support for RTW has shown to be successful and cost-effective for persons with musculoskeletal disorders [36]. However, to the best of our knowledge, no evidence-based digital support exists that improves sustainable RTW for persons with chronic pain and their employers. To fill this gap in knowledge, the aim of this study is to develop a digital support application with evidence-based content that includes a biopsychosocial perspective on chronic pain for sustainable RTW for persons with chronic pain and their employers: SWEPPE (Sustainable Worker Digital Support for Persons With Chronic Pain and Their Employers).

## Methods

### Study Design

**Figure 1:**
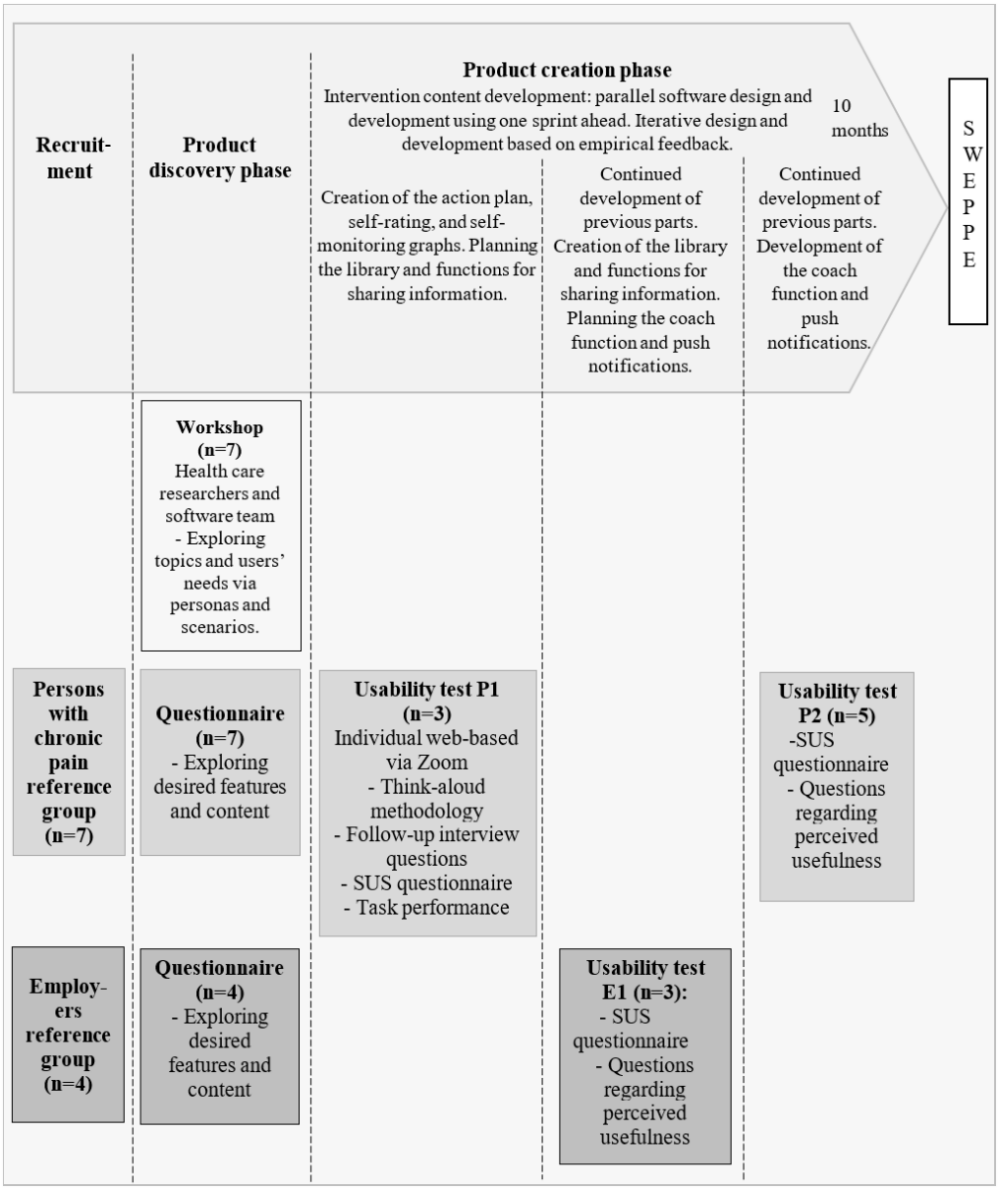
Flowchart of practices and data collection during the development process to create SWEPPE (Sustainable Worker Digital Support for Persons With Chronic Pain and Their Employers). Principles guiding the process: (1) separate product discovery and product creation phases; (2) iterative design and development with empirical feedback to revise designs in the next step; (3) parallel design and development activities using one sprint ahead; (4) continuous involvement of users via reference groups through the process; (5) artifact-mediated communication via user personas and scenarios. SUS: System Usability Scale.

In this study, a user-centered agile design [30] was used. Five principles guided the process [30]: (1) separate product discovery and product creation phases; (2) iterative design and development with empirical feedback to revise designs in the next step; (3) parallel design and development activities using one sprint ahead; (4) continuous involvement of users via reference groups; and (5) artifact-mediated communication via user personas and scenarios (Figure 1).

The multidisciplinary project team consisted of health care researchers, a user representative, and a software team (Table 1).

**Table 1:** Description of the multidisciplinary project team.

Grouping	Total, n (%)	Pain expertise, n (%)	Return-to-work expertise, n (%)	Licensed health care providers, n (%)	Electronic apps expertise, n (%)
Health care researchers^a^	3 (100)	2 (67)	2 (67)	3 (100)	0 (0)
User representative^b^	1 (100)	1 (100)	0 (0)	0 (0)	0 (0)
Software team^c^	5 (100)	0 (0)	0 (0)	0 (0)	5 (100)

^a^PhD occupational therapist.

^b^A research partner from the Swedish Rheumatism Association.

^c^Including user experience design, back-end and front-end development, and project management.

The development process was led by a senior researcher (MB) with extensive experience with RTW, chronic pain, and occupational therapy. Two reference groups representing the end users—patients with chronic pain and their employers—were recruited for the development process. The initial product discovery phase consisted of exploring the users’ needs and wishes about the functions and contents of SWEPPE. The second product creation phase involved design, development, and usability testing of SWEPPE. Mixed methods were applied to collect quantitative and qualitative data for early input and feedback from the users throughout the process.

### Ethics Approval

The Swedish Ethical Review Board approved the study (Dnr 2020-01593).

### Participants in the Reference Groups

Participants in the reference groups were recruited using a relevance sampling strategy [37] conducted at a pain and rehabilitation clinic in southern Sweden. For persons with chronic pain, the following inclusion criteria were used: employed, participation in an interdisciplinary pain rehabilitation program (IPRP) within the last 2 years, and interest in participating in the development of the application. For the employers, the following inclusion criteria were used: experience with an employee who had chronic pain and interest in participating in the development of the application. A total of 12 persons with chronic pain met the inclusion criteria and were invited to the study by email. The invitation contained information about the study, and a telephone follow-up conversation was conducted after approximately 1 week. Of these 12 persons, 4 (33%) did not respond to the invitation or follow-up call, and 1 (8%) declined participation. Thus, of the 12 persons invited, 7 (58%)—4 women and 3 men—with a mean age of 45 (SD 9; range 36-60) years provided informed consent and were included in the study (Table 2).

**Table 2:** Background data on the persons with chronic pain participating in the reference group (n=7).

Characteristic		Values, n (%)
**Reported years living with pain**		
	0-7	3 (43)
	8-14	3 (43)
	>15	1 (14)
**Types of pain**		
	Back or neck pain	4 (57)
	Nerve pain or neuropathic pain	1 (14)
	Fibromyalgia	2 (29)
	Other^a^	3 (43)
**Employment status**		
	Working or studying full-time	4 (57)
	Working or studying part-time	2 (29)
	Sick leave	1 (14)

^a^Leg pain, migraine, and Horton disease.

The mean time since participation in IPRP was 10 (SD 5; range 4-19) months. Of the 7 participants, 1 (14%) had experienced a large degree of support during RTW after IPRP, and 6 (86%) had experienced some support from different stakeholders (employer, health care, or the social insurance agency). A total of 10 employers who previously had been involved in an RTW process for an employee with chronic pain at the rehabilitation clinic were invited to the study in a similar way as the persons with chronic pain. Of these 10 employers, 3 (30%) were not possible to reach and 3 (30%) declined participation in the study owing to lack of time. Finally, of the 10 employers invited, the 4 (40%)—3 men and 1 woman—who agreed to participate in the study were from private and public areas of the labor market (school and education, services and sales, building and manufacturing, or machine and transportation).

### Practices to Develop SWEPPE

#### Overview

Practices such as workshop and usability testing were applied during the different phases of the development of SWEPPE (Figure 1). The reference group of persons with chronic pain participated in 2 usability tests, and the employers participated in 1.

#### Workshop

User personas and two scenarios (*using SWEPPE* and *being back at work*) were developed based on previous research and clinical expertise with persons with chronic pain and other stakeholders. The user personas and scenarios were used in a workshop where 2 health care researchers and the software team verbally and visually presented information about different types of users [38] and about how to bring the needs of the persons with chronic pain and their employers into the development process. For each scenario, brainstorming was performed about what a user persona might think, feel, do, and say in a given situation.

#### Usability Testing

The first usability test was performed with the reference groups of persons with chronic pain (Figure 1, P1) and consisted of both formative and summative testing [39], where screen layouts with partial functionality were used. The test sessions were conducted on the web via Zoom (Zoom Video Communications) meetings owing to the COVID-19 pandemic. Before the first usability test, a pilot test conducted with a person not involved in the project was performed, which led to minor adjustments of the test situation. Then, 3 participants from the reference group of persons with chronic pain participated in individual usability testing sessions. The tests were led by the user experience designer, and the first author (CT) participated as an observer and took notes. During the tests, the participants were given tasks to perform and were asked to verbalize their experience—that is, a think-aloud methodology [40] was applied. All meetings were recorded and shared digitally with 3 members of the software team, who also took notes as part of the think-aloud methodology to aid the development process.

The usability test with the reference group of employers (Figure 1, E1) was performed on a functioning web application. Then, 2 fictional *employees* were created based on the user personas used in the workshop. In addition, 2 researchers acting as these fictional *employees* created accounts in the SWEPPE smartphone app and invited their *employers* in the reference group to access the web application on their own computer. In the web application, the employers could access the library and follow the goals and self-reported data of their employees. The employers received updated information regarding progress from the employees for 3 weeks.

The second usability test with the reference group of persons with chronic pain (Figure 1, P2) was performed on a functioning smartphone app. The persons with chronic pain downloaded and tested SWEPPE at home on their own smartphone for 2 weeks.

### Data Collection

#### Overview

Data were collected from the workshop, digital questionnaires, and usability testing during the development of SWEPPE (Figure 1).

#### Workshop

The workshop generated 2 empathy maps [41] that included short statements of what the user personas might think, feel, do, and say in the given scenarios. These maps were used for the identification of topics, questions, or needs to be considered while developing SWEPPE.

#### Questionnaire

The questionnaire focused on exploring the participants’ experiences of using smartphone apps and suggestions for the functions and content of SWEPPE. The participants in the reference groups were asked to rate the importance of the different proposed functions on a 10-point scale ranging from 1 (not important) to 10 (very important). The proposed functions were based on aspects identified as strengths in previous research, such as setting a goal related to RTW [42-44], the possibility to monitor health status [12], access to a knowledge base about pain and positive examples of RTW [18], digital coaching, and access to frequently asked questions or stories of persons with chronic pain [18,36,42,45].

#### Usability Testing

During the web-based usability test with the persons with chronic pain (P1), data were collected in several steps. First, notes were taken by the first author (CT) and 2 or 3 members of the project team during the think-aloud process regarding what the person said and did when performing the assignments in the SWEPPE prototype. If the test persons were silent, they were prompted by the test leader with questions such as “What are you thinking right now?” Second, at the end of each usability test, the participants were asked open-ended questions regarding the overall impression of SWEPPE: positive or negative functions and content of SWEPPE, what was missing or could be improved, how they would describe SWEPPE to a colleague or friend, and their opinion on how the SWEPPE prototype would be helpful in RTW. Third, after the usability tests, the participants were asked to fill out the System Usability Scale (SUS) questionnaire [46,47] for the global usability assessment of the SWEPPE prototype. The SUS consists of 10 items rated on a 5-point scale ranging from 1 (strongly disagree) to 5 (strongly agree). A total SUS score was calculated, ranging from 0 to 100 (higher scores represent better usability) [46]. A total SUS score >70 represents good usability [48]. Fourth, task performance [39] was assessed during the first usability test where the number of correctly completed tasks was registered by the first author (CT).

After usability tests P2 and E1, both reference groups received the SUS questionnaire and follow-up questions regarding the functions in SWEPPE. The participants were asked to rate the perceived usefulness of different parts of SWEPPE as a support on a scale ranging from 0 (no support) to 100 (maximum support). They were also given the opportunity to comment on the functions and content of SWEPPE—for example, what was missing or could be improved and how they would describe SWEPPE to a colleague or friend.

### Data Analysis

Quantitative data from the surveys and the SUS questionnaire were summarized and analyzed using descriptive statistics. The qualitative data gathered in the empathy maps were summarized and grouped based on topics, questions, and needs to address in the development of SWEPPE.

The notes collected during the think-aloud methodology and data from the open questions in the first usability test with the reference group of persons with chronic pain were analyzed using Instant data analysis [40]. Instant data analysis was performed after each of the usability test sessions, where the test leader, the observer (CT), and 3 members of the software team participated in a Zoom meeting to discuss their notes and the usability problems that had been identified. All usability issues were written down and sorted into groups based on the assignments performed in the prototype: create an account, set a goal related to RTW, review the action plan, finish settings, register daily health status, or follow progress in the overview. The identified usability problems were then discussed by the whole project team and were used to guide adjustments to the SWEPPE prototype before finalizing the application.

## Results

### Product Discovery Phase

The workshop with the project group generated topics, questions, or needs considered during the development process of SWEPPE (Table 3).

Results from the questionnaire about the desired content and functions of SWEPPE showed that maintaining motivation and following progress were of great importance for both reference groups (Tables 4 and 5).

**Table 3:** Identified topics, questions, or needs during the workshop with the project group and empathy mapping of user persona Carina for different scenarios and how these were addressed in SWEPPE^a^.

Scenario and identified topics, questions, and needs to consider in the development process			Addressed in SWEPPE
**Being back at work**			
	Will I manage? Do I have the skills needed?	Goal setting, self-monitoring, and overview to support insights about one’s capacity.	
	Will I get the support I need from the employer?	Identify support wanted from the employer and possibility to share with the employer.	
	Manage balance between work and leisure.	Goal setting, self-monitoring, and overview for feedback.	
	Find a daily routine.	Self-monitoring and overview.	
	Learning new ways.	Library, self-monitoring, and overview.	
	Apply strategies learned during rehabilitation.	Identify barriers to RTW^b^ and strategies to handle them; self-monitoring.	
**Using SWEPPE**			
	How will SWEPPE help me RTW?	Overarching question guiding the general design of all the functions in SWEPPE.	
	Using SWEPPE must be quick and easy.	General design of SWEPPE application as quick and easy to use and demanding low cognitive load.	
	Difficult at first when I started.	General design of SWEPPE when creating and setting up a new account.	
	Uncertain about what data the employer can see in SWEPPE.	General design of SWEPPE with easy access to information the user wants to share with the employer.	
	Feeling guilty if not using SWEPPE every day.	Are data presented in the overview in a useful way even if data are missing?	
	Proud and happy about her progress.	Design of overview for easy visualization of progress.	

^a^SWEPPE: Sustainable Worker Digital Support for Persons With Chronic Pain and Their Employers.

^b^RTW: return to work.

**Table 4:** Results from the initial survey with persons with chronic pain (n=7) regarding the desired content and functions of SWEPPE^a^.

Questions, desired content, and topics of interest			Rating of importance, median (IQR)
**An application for people with chronic pain and their employer as support for return to work (SWEPPE) would be interesting**			
	To keep me motivated	10 (9-10)	
	To follow and focus on my progress	9 (7-10)	
	To keep track of my health status	8 (8-10)	
	To keep track of my work situation	8 (4-10)	
	To get inspiration from others	8 (59)	
**Important information to know about the application**			
	Security details or privacy information	10 (8-10)	
	How to optimize usability	8 (8-10)	
	Where information in the app comes from	8 (8-10)	
**Desired content or topics of information in SWEPPE**			
	Pain and coping with pain	10 (9-10)	
	Stress and coping	9 (8-10)	
	Work and work ability	9 (7-10)	
	Ergonomics	9 (6-10)	
	Thoughts and feelings	8 (8-9)	
	Balance in daily activities	8 (8-10)	
	Coping during hard times	8 (8-9)	
	Healthy lifestyle	8 (7-10)	
	Others’ experiences of coping with chronic pain	8 (7-9)	
	Workplace adaptation	8 (8-9)	
	Communication, relations, social support	7 (6-9)	
**Desired functions in SWEPPE**			
	Setting goals	9 (8-10)	
	Communicate with a coach	9 (7-10)	
	FAQ (frequently asked questions) available	8 (7-9)	
	Tips on workplace adaptation	8 (7-9)	
	Communicate information with my employer	8 (7-9)	
**Important functions SWEPPE should have**			
	Push notifications	9 (2-10)	
	Adaptive functions	8 (7-9)	
	Adaptive design	8 (6-8)	
	Download information	6 (5-10)	
**Preferred health aspects to record in SWEPPE or receive information about from employee**			
	Pain	10 (10-10)	
	Sleep	10 (8-10)	
	Physical activity	10 (8-10)	
	Work situation	10 (6-10)	
	Balanced life situation	8 (7-10)	
	Workload	7 (5-9)	

^a^SWEPPE: Sustainable Worker Digital Support for Persons With Chronic Pain and Their Employers.

**Table 5:** Results from the initial survey with employers (n=4) regarding the desired content and functions of SWEPPE^a^.

Questions, desired content, and topics of interest			Rating of importance, median (IQR)
**An application for people with chronic pain and their employer as support for RTW^b^ (SWEPPE) would be interesting**			
	To motivate and support the employee	9.5 (9-10)	
	To follow the employee’s progress	9.5 (9-10)	
	To receive information about my responsibility as an employer	9.5 (9-10)	
	To receive tips on adaptation of the work situation	9.5 (9-10)	
	To follow the employee’s work situation	8.5 (8-9)	
	To follow the employee’s health status	8.5 (8-9)	
	To receive information about chronic pain	7.5 (7-9)	
	To get inspiration from others	6.5 (6-8)	
**Important information to know about the application**			
	How to optimize usability	8.5 (8-9)	
	Where information in the app comes from	8.5 (8-9)	
	Security details or privacy information	8.5 (8-9)	
**Desired content or topics of information in SWEPPE**			
	Work and work ability	9.5 (9-10)	
	Ergonomics	9 (8-9)	
	Information about my responsibility as an employer	9 (9-10)	
	Stress and coping	9 (9-9)	
	About pain and coping with pain	9 (9-9)	
	Workplace adaptation	9 (8-9)	
	Balance in daily activities	8.5 (8-9)	
	Coping during hard times	8 (7-9)	
	Communication, relations, social support	8 (7-8)	
	Thoughts and feelings	8 (6-9)	
	Healthy lifestyle	7 (5-9)	
	Others’ experiences of coping with chronic pain	6 (4-8)	
**Desired functions in SWEPPE**			
	Receive information about the employee’s goals	9 (9-9)	
	Tips on workplace adaptation	9 (9-9)	
	FAQ^c^ available	7 (6-9)	
**Important functions SWEPPE should have**			
	Adaptive design	8 (7-9)	
	Adaptive functions	6.5 (6-8)	
	Download information	6.5 (6-7)	
	Push notifications	4.5 (2-7)	
**Preferred health aspects to record in SWEPPE or receive information about from employee**			
	Work situation	10 (10-10)	
	Workload	10 (10-10)	
	Pain	9.5 (9-10)	
	Physical activity	9 (9-9)	
	Sleep	8.5 (8-9)	
	Balanced life situation	8.5 (7-9)	

^a^SWEPPE: Sustainable Worker Digital Support for Persons With Chronic Pain and Their Employers.

^b^RTW: return to work.

^C^FAQ: frequently asked questions.

For the persons with chronic pain, the opportunity to keep track of their health status and work situation was also important. Employers wanted information about their responsibility and suggestions for adapting the work situation. Both reference groups preferred getting feedback through graphs showing changes over time. Persons with chronic pain wanted to use SWEPPE on their smartphone, and most (4/7, 57%) reported wanting to use SWEPPE daily. The employers had a diverse view of how often they would use SWEPPE. An employer wanted to use SWEPPE when needed, another weekly, and another monthly. Most of the persons with chronic pain preferred recording pain (5/7, 71%) and sleep (4/7, 57%) daily and physical activity (4/7, 57%), balanced life situation (5/7, 71%), and work situation (4/7, 57%) weekly. The opinion among the persons with chronic pain about push notifications was mixed: push notifications were rated as an important function (Tables 4 and 5), but a majority (4/7, 57%) did not want them included in the SWEPPE application. However, most participants noted that their acceptance of push notifications would depend on the available settings. The other characteristics that the reference groups rated as important were compatibility with a smartphone and ease of use. The results from the questionnaire were used to prioritize the functions and development of SWEPPE.

### Product Creation Phase

The initial development of SWEPPE, based on the first survey of persons with chronic pain, focused on three aspects: (1) the action plan, where the users assess their work ability, set a goal related to RTW, identify barriers to RTW, develop strategies to handle barriers, and identify support wanted from the employer; (2) self-rating, where the users register daily health status and work situation; and (3) self-monitoring graphs, where the users follow their progress and receive feedback to keep them motivated.

These 3 aspects were developed and tested along with the overall design (eg, colors and layout) in the first usability test (P1). The participants in usability test P1 were in general positive to the prototype and experienced it as relevant, quick, and easy to use. They stressed the importance of SWEPPE not demanding too much of them cognitively. They described SWEPPE as a tool to help them stay motivated and learn more about themselves and their pain. The task performance rate was high (Table 6).

**Figure 2:**
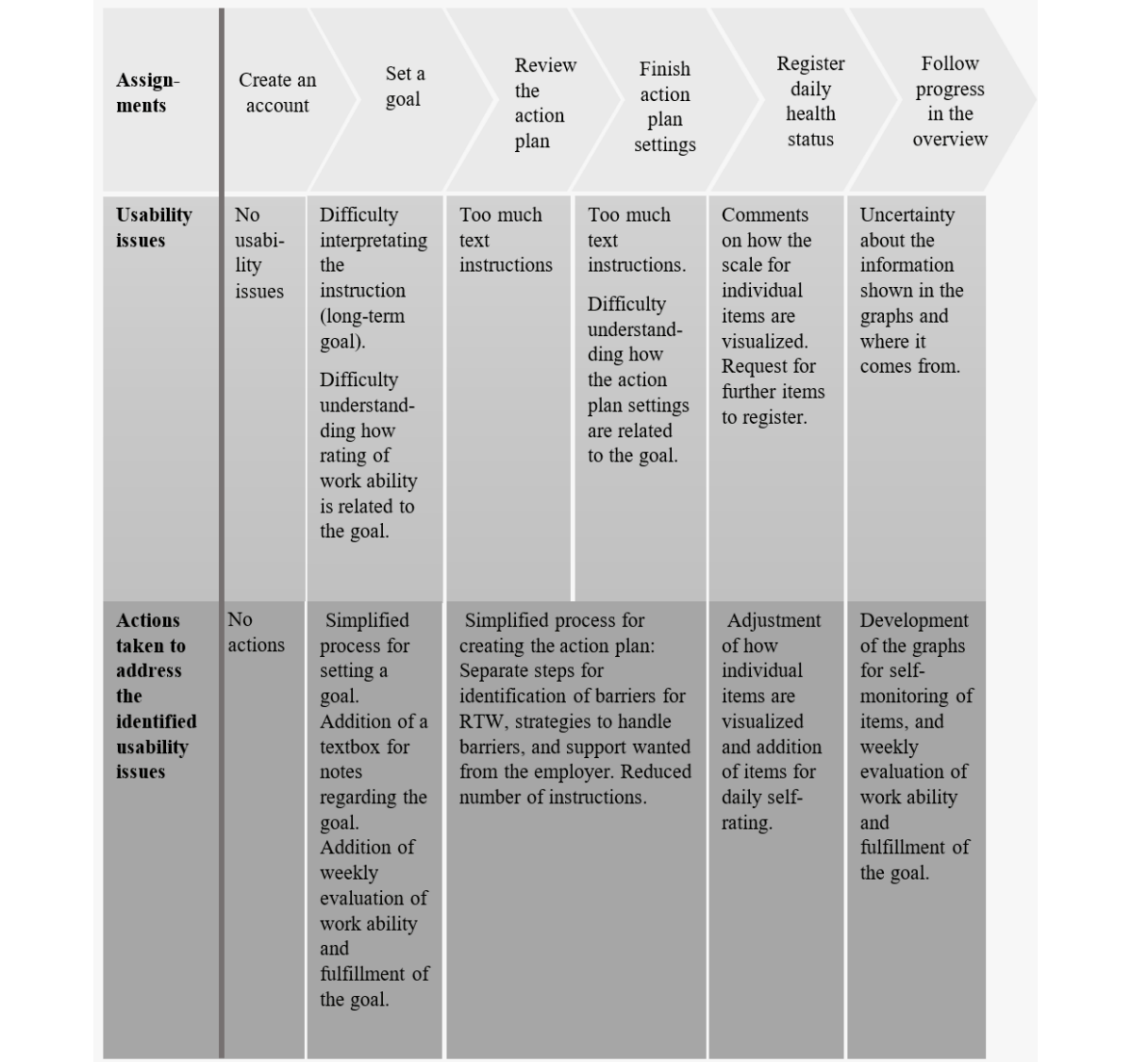
Overview of the assignments in usability test P1 performed by participants (n=3) from the reference group of persons with chronic pain, the usability issues identified during the think-aloud methodology, and how these issues were addressed in the continued development process. RTW: return to work.

Some usability problems were identified using the think-aloud methodology and were addressed in the continued development process (Figure 2).

**Table 6:** Results from usability testing P1 and P2 with the persons with chronic pain and E1 with the employers. Data collection from assessment of task performance and questionnaires.

Time points		Usability test: P1 persons with chronic pain (n=3)	Usability test: E1 employers (n=3)	Usability test: P2 persons with chronic pain (n=6)
**Task performance^a^, n (%)**				
	Create an account	3 (100)	N/A^b^	N/A
	Set a goal	3 (100)	N/A	N/A
	Review the action plan	3 (100)	N/A	N/A
	Finish action plan settings	3 (100)	N/A	N/A
	Register daily health status	3 (100)	N/A	N/A
	Follow progress in the overview	3 (100)	N/A	N/A
**SUS^c^ questionnaire, median (IQR)**				
	SUS score point^d^	95 (94-98)	88 (72-89)	86.5 (77-94)
**Employers perceived usefulness^e^ of receiving information, median (IQR)**				
	About the employee’s work-related goal	N/A	74 (58.5-83.5)	N/A
	About barriers for RTW^f^ identified by the employee	N/A	71 (61.5-85.5)	N/A
	About strategies identified by the employee	N/A	46 (32.5-59.5)	N/A
	About support wanted from the employer	N/A	73 (67.5-86.5)	N/A
	To follow the employee’s progress in a graph	N/A	74 (70.5-87)	N/A
	From the library	N/A	50 (31-62)	N/A
	To be reminded of using SWEPPE^g^	N/A	100 (55-100)	N/A
**Persons with chronic pains perceived usefulness of SWEPPE, median (IQR)**				
	Setting a work-related goal and following the progress	N/A	N/A	81 (53.3-92.3)
	Identifying barriers and strategies for RTW	N/A	N/A	68 (53-90.5)
	Sharing information with the employer	N/A	N/A	53.5 (28.3-60.8)
	Self-monitoring health aspects and getting an overview	N/A	N/A	80 (56-88.3)
	Using the library	N/A	N/A	60.5 (54-75.3)
	Asking questions and receiving answers from the coach	N/A	N/A	47 (41.5-69)
	Getting reminders of daily self-rating of health aspects and weekly evaluation of goal fulfillment	N/A	N/A	85.5 (70.8-95.8)

^a^Number of correctly completed tasks.

^b^N/A: not applicable.

^c^SUS: System Usability Scale.

^d^SUS score points range from 0 to 100, where higher scores represent better usability.

^e^Rated on a visual analogue scale ranging from 0 (no support) to 100 (maximum support).

^f^RTW: return to work.

^g^SWEPPE: Sustainable Worker Digital Support for Persons With Chronic Pain and Their Employers.

The process of setting a goal related to RTW and creating the action plan was simplified, and questions for assessment of goal fulfillment were added: further improvements of self-rating of health aspects were made with addition and development of items, and separate graphs for self-monitoring of health aspects and goals were developed. Continuous adjustments and refinements of the functions based on the usability test P1 were made, and further parts of SWEPPE (eg, the library, the coach function, and the user profile for the employer) were developed.

The version of SWEPPE evaluated in usability tests E1 and P2 consisted of a fully functioning web application for the employers and a smartphone app for the persons with chronic pain. The employers perceived receiving information about the employee’s goal related to RTW, barriers for RTW, support wanted from the employer, and the graph to follow the employee’s progress as the most useful functions in SWEPPE (Table 6). The persons with chronic pain participating in test P2 rated self-monitoring, setting a goal related to RTW, and identifying barriers and strategies as the most useful functions. Overall, the participants found SWEPPE to be helpful. For example, they thought the application was safe, provided relevant information, and would be good for many people with chronic pain. Regarding usability, the median SUS scores of the employers and persons with chronic pain were high (median 88 and 86.5, respectively; Table 6). SWEPPE was deemed to be logical and easy to use with an appealing interface. The participants in tests E1 and P2 also provided several comments regarding the different functions in SWEPPE, and the employers provided suggestions for ways to improve the application (Table 7).

**Table 7:** Overview of SWEPPE^a^ and the modules and their content evaluated in the usability tests with the employers (test E1, n=3) and persons with chronic pain (test P2, n=5).^b^

Module in SWEPPE	Description of content	Comments from participants in tests E1 and P2
The action plan^c^	Goal setting regarding work; identification of barriers to RTW, strategies to handle these barriers, and support wanted from the employer; and weekly evaluation of work ability and fulfillment of the goal	“Good help to set a goal with the suggestions and getting a summary in the overview” [P] “Having a goal makes it easier to do the little extra to fulfil your wishes” [P] “By identifying the barriers, it is easier for you to find strategies to work around them. Otherwise, it is easy to end up with bad habits and you don’t know why” [P] “I like the suggestions for strategies because many might not even think about it” [P]
Daily self-rating^c^	Self-rating of health and psychosocial aspects, work situation, and strategies	“A very good part” [P] “Good to be able to choose what health aspects to monitor” [P]
Self-monitoring graphs^c^	Graphs for self-monitoring health aspects, work ability, and progress toward the goal over time	“Good with the summary in a graph” [P] “It is easier to capture trends like not doing exercise when you have a lot of other things to do. Then you get the information in black and white that you have skipped exercise too many days and you can follow the pain curve which due to lack of exercise is getting worse” [P] “To follow pain, stress and physical activity would help me a lot. It can help to do more exercise and it gives you a great summary if the activity helps for the pain” [P]
The coach^c^	Opportunity to ask a question and receive a written answer from a coach	“Superb function to be able to get help via the app” [P] “Surely good if you need support in some way like how to handle your employer” [P]
The library^c,d^	Knowledge database developed based on previous research with information (texts, films, and audio clips) that reflects a biopsychosocial perspective regarding chronic pain, physical activity, managing the situation, activity pacing, balance in daily life, sleep, workplace adaptations, tools for dialogue, and answers from the coach on common questions	“Good texts and films. If only the employer has the time and will to learn there is a lot of good material in the app. Not only for the employer but also for me” [P] “This would have been useful for me earlier [in the RTW process]” [P] “I liked the library. A lot of good information” [P] “Gathered information is always good” [E]
Shared information with the employer^c,d^	The person with chronic pain can give the employer access to the library and share information from the action plan and the graph for monitoring work ability and goal fulfillment in SWEPPE, and the employer receives the information from the parts of the action plan the employee has chosen to share; if the employee does not want to share any information from the action plan, the employer still has access to the library	“Good and perspicuous arrangement of goal, barriers, strategies and wanted support” [E] “Can meetings be visualized? Reconciliation meetings with the occupational health care services is an important basis that would be good to see in the graph” [E] “It would be valuable to follow up strategies from the employee and employer that have not given results, that is changes in strategies and support wanted from the employer during rehabilitation. What has given results in the right direction and what has not” [E] “Clearer start and goal of the weekly evaluations, it would add value if you could register concrete actions to follow up” [E] “A simple platform for quickly finding gathered information and the employee’s progress” [E] “This is not applicable for me right now but if I would increase my working time, it would be very good to involve the employer. I think SWEPPE would be good both for me and for my employer as long as the employer has the will. The formulation in the app is clear and I think it would make communication between the employer and the employee easier” [P] “It can be difficult to get you employer involved but with SWEPPE it can be easier for the employer to see if there is a negative trend. Unfortunately, I don’t think everybody would dare to share with their employer and some employers will probably not be so engaged or even look in SWEPPE” [P] “It’s good to be able to give you employer insights about how you feel and you choose how much you want to share” [P]

^a^SWEPPE: Sustainable Worker Digital Support for Persons With Chronic Pain and Their Employers.

^b^These modules also constituted the final version of SWEPPE.

^c^Accessed by persons with chronic pain via the smartphone app.

^d^Accessed by the employers via the web application.

**Figure 3:**
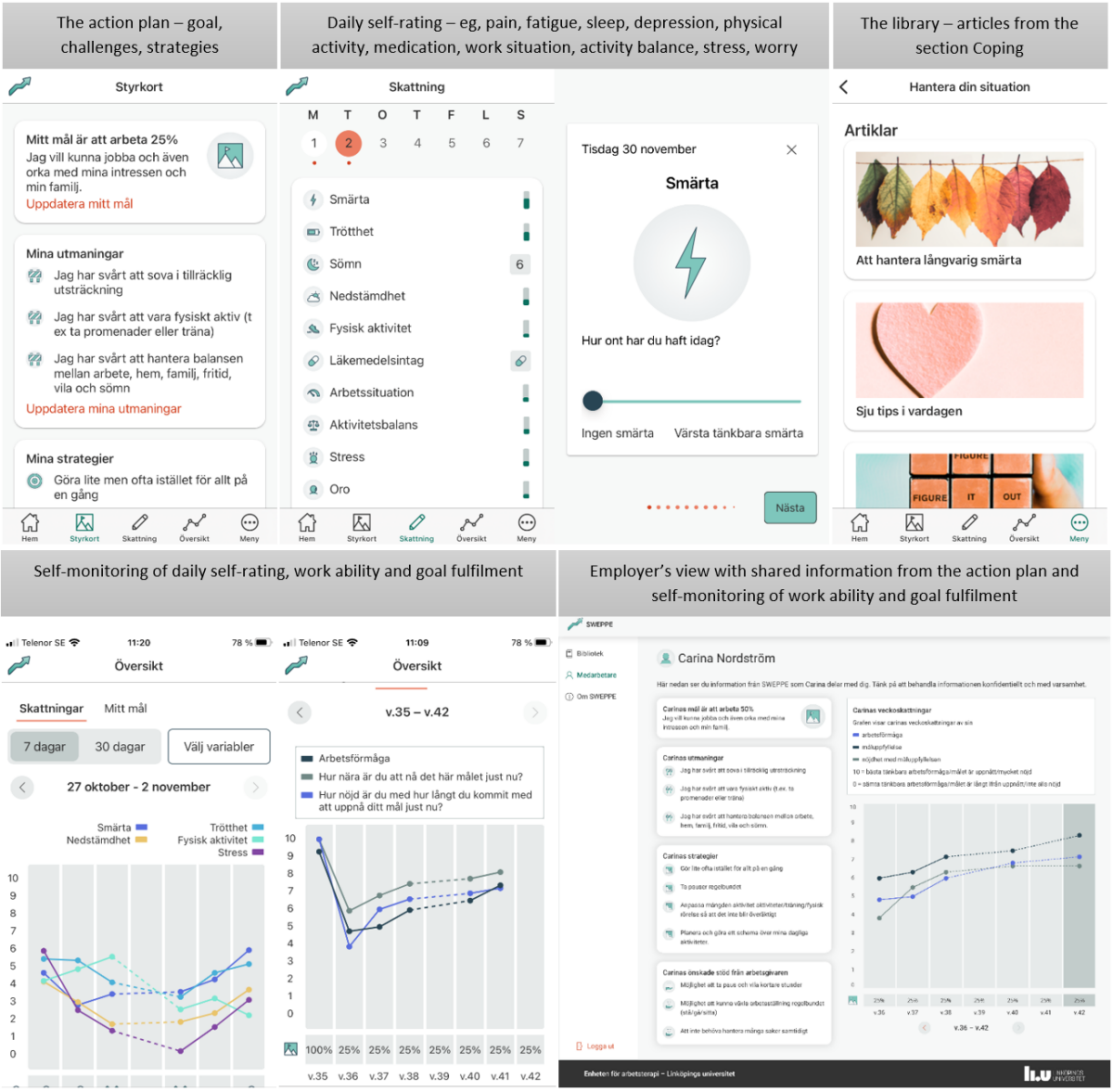
Screenshots from the final version of SWEPPE. SWEPPE is available in Swedish. Top row from left to right: the action plan (Styrkort), daily self-registration overview (Skattning) and rating of pain (Smärta), one of the library sections (Hantera din situation). Bottom row: self-monitoring (Översikt) of health aspects, work ability and goal fulfilment, employer’s view of shared information from the employee.

The final version of SWEPPE consisted of all the modules presented in Table 7. In the final version, the content of the action plan (the goal and identifying strategies and support wanted from the employer) can be individualized by the employee. The user is presented with different options (strategies or needs) to choose from, but these can be modified, and the user can also create their own options in the app. For daily self-rating of health aspects, the user is given the possibility to self-monitor not only bio-related aspects such as fatigue and pain but also psychosocial aspects such as work situation and activity balance. For daily self-rating of, for example, pain, a slider for a visual analog scale was used, ranging from 0 (no pain) to 10 (worst imaginable pain). The value is not indicated on the screen when the user is performing daily self-rating but is presented in the self-monitoring graph. Screenshots from SWEPPE are presented in Figure 3.

## Discussion

### Principal Findings

This study describes the development of SWEPPE, a digital support application for sustainable RTW for persons with chronic pain and their employers. SWEPPE was developed with a UCD agile approach [30], where the foundation was evidence-based knowledge of chronic pain reflecting a biopsychosocial perspective and RTW in combination with involvement of the end users during the development process. To our knowledge, SWEPPE is the first eHealth intervention for both patients with chronic pain and their employers in supporting a sustainable RTW.

SWEPPE was developed by a multidisciplinary project team using a combination of the 5 principles of UCD agile design [30]. First, the separate product discovery and product creation phases provided relevant content and functions initially discovered through a workshop and a questionnaire, which led to the development of a low-fidelity prototype [39] that was tested and constituted the foundation for the product creation phase. Second, the iterative design and development used feedback from the reference groups at different stages to revise the design. Third, the software team used parallel design and development activities using one sprint ahead with scheduling and organization of the development in 2-week sprints. Fourth, continuous involvement of the end users throughout the process was ensured by using 2 reference groups who participated in questionnaires and usability testing and a user representative as part of the project team, which all provided valuable information. Fifth, artifact-mediated communication was used for the user personas and scenarios in the workshop and for the employers in the usability test. Applying these principles in combination with the competencies of the multidisciplinary project team ensured performance of systematic evaluation and development of a product relevant for the users’ context. In this study, participants with chronic pain as well as the user representative emphasized the importance of presenting information in an easy, understandable way that did not require a large cognitive load. This finding is consistent with findings by Ledel Solem et al [15], where participating patients preferred a simpler presentation of content rather than gamifying design elements, as these could be challenging to use when experiencing chronic pain. The results from this study show that SWEPPE was deemed easy to use, which has been identified as a facilitator for using eHealth applications by persons with chronic pain [17].

### Supporting Self-management

This study shows that SWEPPE has the potential to be a valuable tool for supporting the individual in self-management of chronic pain during the RTW process. Web-based applications or smartphone apps can be easily accessed and enable persons with chronic pain manage their condition [49] and reduce pain interference [50]. As self-management and empowerment have been identified as important parts of successful eHealth interventions [51], SWEPPE was developed to target the lack of support experienced by persons with chronic pain after finishing a rehabilitation program and where the RTW process continues [7]. Self-monitoring of health aspects was an important part to include in SWEPPE, as it is a common strategy for self-management among persons with chronic pain [17,20]. The daily self-rating in SWEPPE generated data presented in graphs for self-monitoring, a function the participants with chronic pain perceived as useful. Individuals are different in their tolerance of pain and the daily self-rating of pain in SWEPPE reflects the individual’s subjective experience. The user can also choose which and how many of the biopsychosocial aspects available in SWEPPE to monitor, based on the relevance for the individual’s specific situation. Self-monitoring in SWEPPE provides the user with feedback, which can contribute to learning about health aspects in relation to actions and behaviors in daily life [52]. Patients’ understanding of their own self-monitoring data involves perception of the information, making inferences, and using these to change their daily activities [22], which can give them a sense of control and motivation to continue using self-management strategies [23].

Pain education is also a common part of self-management and can be related to the neuroscience of pain, medication, stress, depression, and sleep management [19]. In SWEPPE, the library was developed to provide easily accessed information about chronic pain based on a biopsychosocial perspective. The content in the library was intended to support both the employee and the employer by contributing to an increased understanding of the need to take the whole life situation into account when planning for RTW. The library was especially important for employers who wanted knowledge about how chronic pain might affect work ability and how they can support the employee during RTW [7]. Providing information through computer applications and smartphone apps has been shown to improve the level of knowledge, and the effectiveness can increase by 78% when also using at least one push notification a week [53]. In SWEPPE, a randomly selected text from the library was suggested once a week for the user in the smartphone app to inspire continuous reading. Although the library was perceived as useful by both reference groups, it was not rated as useful as other functions in SWEPPE. According to Timmers et al [53], the timing of information is crucial, as patients need to receive the right information at the right time. In this study, persons with chronic pain found that the information in the library would have been useful for them earlier in their RTW process, indicating the potential and need of SWEPPE in a clinical setting, when the users are starting their RTW process.

Regarding the dynamics of the employee-employer relationship, the participants in both reference groups were in general positive to the function of sharing information in SWEPPE to facilitate collaboration and communication between the employee and the employer. SWEPPE was built to be al tool for providing the employer with information but without the employee having to educate the employer regarding chronic pain and its consequences for work. Instead, by using SWEPPE, the employee can invite the employer and give access to the library and decide what information to share from their action plan. If the employer is willing to engage in the process and use the provided information, this could increase the employer’s ability to support the employee. The issue of employees not wanting or daring to share information with their employer was raised by some of the persons with chronic pain during usability testing. An important feature of SWEPPE was to make the employee in charge of what information to share, when and with whom to avoid the employer from using the app for control or pressure. In this study, usability tests P2 and E1 revealed the persons with chronic pain perceived the function sharing information as slightly less useful compared with the employers and compared with other functions in SWEPPE. This was due to the participating persons with chronic pain having come too far in their RTW process and commented on this function as being more useful earlier in the process. This stresses the importance of getting the user’s context correct in the development of digital solutions [34]. Therefore, the usefulness of sharing information also needs to be tested further in a clinical setting to study the interplay between the employer and the employee.

### Strength, Limitations, and Future Directions

There are some limitations of this study that need to be considered. SWEPPE was developed in Sweden, where the employers are prescribed by law to take actions to adapt the workplace to the individual’s capacity and thereby enable the employee to RTW or stay at work. However, the rights and responsibilities of employees and employers vary among countries, and the usefulness of an app such as SWEPPE may depend on the societal system.

The number of persons in the reference group of persons with chronic pain was small and might not be representative of the whole population of persons with chronic pain. However, the participants are representative of the targeted users of SWEPPE (ie, persons with chronic pain who have participated in an IPRP and who have experiences with the RTW process). The persons with chronic pain participating in this study had come further in their RTW process than the intended users of SWEPPE. This was regarded as a strength of the study, as the participants had the experience and possibility to reflect on their needs during the RTW process and could acknowledge that SWEPPE had been useful for them earlier in the RTW process. As a result, there were lower ratings of some of the functions (eg, coach and sharing information), as these were not needed in the participants’ present situation. There were also few employers participating in this study, and recruitment of employers to the reference group was more difficult, as they were experiencing a lot of time pressure. However, having employers involved in the development of SWEPPE was crucial and is a strength of this study, as they play an important role in the RTW process [54]. Overall, the small number of participants in the 2 reference groups contributed with a variety of valuable feedback relevant to the end users.

Another strength of this study was having a user representative as part of the multidisciplinary project team, as the experience-based knowledge provided by a research partner complemented the professional knowledge [55]. The user representative gave valuable feedback during the whole process on ideas, functions, and texts and helped prioritize the suggestions from the reference groups, which validated decisions made during the design and development of SWEPPE.

A strength of this study is also the use of both qualitative and quantitative methods for evaluation and feedback during the development process [56], which gave valuable information for the development of SWEPPE. During the process of creating SWEPPE, it was decided that content, functionality, and design were the most important parts to examine in usability testing and the feedback from the reference groups. Thus, not all the written texts were evaluated in the tests with the reference groups.

Usability testing requires advanced planning and involves several decisions such as selecting the setting, the tasks the user should perform, and the type of data to be collected. In this study, 3 persons with chronic pain participated in the initial usability test (P1) of the low-fidelity prototype. This may have been too few to identify all possible usability issues. It has been suggested that 5 participants are sufficient for usability testing and finding 80% of the usability problems [39]. Still, valuable information was collected during the test that confirmed that the basic structure and content in SWEPPE were in line with the users’ desires and needs. The COVID-19 pandemic also influenced the options regarding testing. For example, it was not possible to conduct the tests during a physical meeting at the university. Doing a usability test on the web via a Zoom meeting might have affected the willingness for some participants to participate in the initial test. People willing to participate in a Zoom meeting might also indicate a selection bias, as these people probably were more comfortable with using technology than people who chose not to participate perhaps because they were intimidated by technology. More participants participated in the usability tests performed on the functioning smartphone apps or web applications tested at home (E1 and P2), which can be a result of the participants feeling more comfortable using their own smartphone or computer in a familiar environment [57]. These tests were performed to validate the nearly finished version of SWEPPE and to collect suggestions for further improvements. A strength of these tests was that none of the participants needed help getting started with SWEPPE.

The results of the development of SWEPPE are positive and highly usable because of the UCD agile approach. However, to investigate its effectiveness, SWEPPE needs to be tested in a clinical setting, initially in a pilot study and then in a randomized clinical trial.

### Conclusions

This study reports the development of a digital support application for persons with chronic pain and their employers. SWEPPE fulfilled the need of support after IPRP with useful functions such as setting a goal related to RTW, identifying barriers and strategies for RTW, self-monitoring, and sharing information between employee and employer. The UCD agile design approach contributed to creating SWEPPE as a relevant and easy-to-use eHealth intervention. Further studies are needed to examine the effectiveness of SWEPPE in a clinical setting.

## Abbreviations

IPRP: interdisciplinary pain rehabilitation program
RTW: return to work
SUS: System Usability Scale
SWEPPE: Sustainable Worker Digital Support for Persons With Chronic Pain and Their Employers
UCD: user-centered design
